# Brain-inspired semantic data augmentation for multi-style images

**DOI:** 10.3389/fnbot.2024.1382406

**Published:** 2024-03-26

**Authors:** Wei Wang, Zhaowei Shang, Chengxing Li

**Affiliations:** College of Computer Science, Chongqing University, Chongqing, China

**Keywords:** data augmentation, deep learning, robust statistics, style transfer, uncertainty modeling, brain-inspired computer vision

## Abstract

Data augmentation is an effective technique for automatically expanding training data in deep learning. Brain-inspired methods are approaches that draw inspiration from the functionality and structure of the human brain and apply these mechanisms and principles to artificial intelligence and computer science. When there is a large style difference between training data and testing data, common data augmentation methods cannot effectively enhance the generalization performance of the deep model. To solve this problem, we improve modeling Domain Shifts with Uncertainty (DSU) and propose a new brain-inspired computer vision image data augmentation method which consists of two key components, namely, *using Robust statistics and controlling the Coefficient of variance for DSU* (RCDSU) and *Feature Data Augmentation* (FeatureDA). RCDSU calculates feature statistics (mean and standard deviation) with robust statistics to weaken the influence of outliers, making the statistics close to the real values and improving the robustness of deep learning models. By controlling the coefficient of variance, RCDSU makes the feature statistics shift with semantic preservation and increases shift range. FeatureDA controls the coefficient of variance similarly to generate the augmented features with semantics unchanged and increase the coverage of augmented features. RCDSU and FeatureDA are proposed to perform style transfer and content transfer in the feature space, and improve the generalization ability of the model at the style and content level respectively. On Photo, Art Painting, Cartoon, and Sketch (PACS) multi-style classification task, RCDSU plus FeatureDA achieves competitive accuracy. After adding Gaussian noise to PACS dataset, RCDSU plus FeatureDA shows strong robustness against outliers. FeatureDA achieves excellent results on CIFAR-100 image classification task. RCDSU plus FeatureDA can be applied as a novel brain-inspired semantic data augmentation method with implicit robot automation which is suitable for datasets with large style differences between training and testing data.

## 1 Introduction

Data augmentation is a strategy to increase the quantity and diversity of limited data, aiming to extract more useful information from limited data and generate value equivalent to more data. It is a technique with implicit robot automation to automatically expand training data. Aiming at the problem of model overfitting in training deep networks (Krizhevsky, 2009; Simonyan and Zisserman, 2014; He et al., 2016; Krizhevsky et al., 2017; Huang et al., 2019), data augmentation methods attempt to solve the problem from the root cause, namely, insufficient training samples (Wang et al., 2019; Liu et al., 2023). Data augmentation is widely used in text classification (Wei and Zou, 2019; Fang et al., 2022; Wu et al., 2022; Dai H. et al., 2023), image denoising (Eckert et al., 2020; Liu et al., 2020; Luo et al., 2021), video recognition (Cauli and Reforgiato Recupero, 2022; Gorpincenko and Mackiewicz, 2022; Kim et al., 2022), etc. In image recognition tasks, there are content-preserving transformations on input samples, such as rotation, horizontal mirroring, cropping and color jittering. Although these augmentation methods are effective, they cannot perform semantic transformations such as changing the background of an object or changing visual angle. The semantics-preserving transformations which preserve class identity can make data augmentation more powerful (Antoniou et al., 2017; Ratner et al., 2017; Bowles et al., 2018). For example, by training a generative adversarial network (GAN) for each class in training set, an infinite number of samples can be sampled from the generator. However, this process is computationally expensive, since both training generative models and inferring them to obtain augmented samples are difficult tasks. In addition, the training process may also be lengthened due to the additional augmented data. Brain-inspired methods are approaches that draw inspiration from the functionality and structure of the human brain and apply these mechanisms and principles to artificial intelligence and computer science (Zendrikov et al., 2023).

When encountering datasets with large style differences between training data and testing data, that is, multi-style datasets, common data augmentation methods cannot effectively enhance the generalization performance of the deep model (Li et al., 2022). Therefore, it is very important to study data augmentation methods for multi-style datasets. In this paper, we propose a brain-inspired computer vision image data augmentation method for multi-style datasets in the feature space with semantic preservation which is highly efficient.

Our approach is motivated from the following three aspects: (1) Existing data augmentation methods such as implicit semantic data augmentation (ISDA) (Wang et al., 2021) and so on mostly augment data by changing the image content without changing the image style. They can work well in situations where there are only content differences but not style differences between training data and testing data, such as CIFAR-10 and CIFAR-100 datasets. However, when there are large style differences between the training data and testing data, such as Photo, Art Painting, Cartoon, and Sketch (PACS) dataset, the common data augmentation methods cannot work well. Modeling Domain Shifts with Uncertainty (DSU) (Li et al., 2022) changes the image style, but it does not change the image content. From the perspective of brain inspiration, we can explore and utilize the structure and functionality of the human brain to improve the performance of data augmentation. For example, when we observe an image, we will pay attention to its content and style, such as a dog with painting style, a cat with sketch style, a car with photo style and so on. Therefore, to improve the diversity of data augmentation results, in the actual application process, we may need to perform both style transfer and content transfer when generating augmented images from original images. Previous studies did not combine style transfer with content transfer. In this paper, we combine content transfer and style transfer by performing style transfer on the feature map, and then performing content transfer on the feature vector learned by the feature extraction network. (2) Real data is often mixed with noise. When the training data is mixed with noise, the model often faces the problem of performance degradation, mainly because the noise will bring outliers, which deviate from the overall distribution. Outliers will interfere with the model, making the model unable to extract key features of the sample, or making the model learn wrong features. In this paper, we calculate feature statistics (mean and standard deviation) with robust statistics to weaken the influence of outliers, making the statistics close to the real values and improving the robustness of deep learning models. (3) From the perspective of brain-inspired computer vision, the distribution of sample data can be regarded as a “spherical space,” which can be regarded as a circle in two-dimensional space and a sphere in three-dimensional space (Jeon et al., 2022). For the convenience of expression, we use “sphere” to refer to the “spherical space” of any dimension. The data points are distributed layer by layer from the center of the sphere outward. Due to different positions, the data augmentation strategies of the sample points at the center of the sphere and the data augmentation strategies of the sample points at the outermost layer of the sphere should be different. However, the existing augmentation method does not consider the spherical distribution characteristics of the sample data, and treats all data equally. In this paper, from the perspective of brain-inspired computer vision, the data augmentation strategy of each point is determined according to the distance between each point and the center point.

According to DSU, it calculates the variance of all feature statistics in a mini-batch, and then uses the variance to generate random shifts to add to the original feature statistics. All feature statistics in a mini-batch share the same variance. However, we think that for all the feature statistics in a mini-batch, when considering their data distribution characteristics, the added shifts of the feature statistics distributed in the center of the group and at the edge of the group should be different. In order to keep the semantics unchanged, the shifts added to the feature statistics distributed at the edge of the group should be slightly smaller and in order to increase the coverage after shifting, the shifts added to the feature statistics near the center of the group should be slightly larger. DSU calculates the mean and variance by channel for each feature map, that is, calculates the mean and variance for all pixel values of each channel. However, this direct calculation of the mean and variance does not take into account the impact of outliers. The appearance of outliers will lead to great deviation in statistical results. In order to reduce the influence of outliers, this paper adopts the method of robust statistics to improve the stability of the model. In this paper, we improve DSU, and obtain the improved brain-inspired computer vision method using Robust statistics and controlling the Coefficient of variance for DSU (RCDSU), which calculates feature mean and standard deviation with robust statistics and controls the coefficient of variance to preserve semantics and increase shift range. According to ISDA, it enhances the generalization ability of the model through implicit semantic data augmentation. It works by computing the covariance of all features for each class, and then for each feature, using the covariance of corresponding class to generate a random shift to add to the original feature. This method needs to use the online algorithm to iteratively update the covariance matrix of each class, which is computationally intensive and the obtained covariance matrix is an estimated value rather than an accurate value most of the time. Therefore, this paper proposes a new augmentation method Feature Data Augmentation (FeatureDA), which calculates the variance of all features in a mini-batch, and then uses the variance to generate a random shift to add to the original feature. In order to keep the semantics unchanged, the shifts added to the features distributed at the edge of the group should be slightly smaller and in order to increase the coverage after shifting, the shifts added to the features near the center of the group should be slightly larger, similar to RCDSU. Our proposed method is simple and effective, and enhances the generalization ability and the stability against outliers of the model. Our brain-inspired computer vision method can be integrated into existing networks without introducing redundant model parameters or loss constraints. Experiments have proved that RCDSU and FeatureDA can improve the generalization ability of the model at the style level and at the content level respectively.

In summary, there are three major contributions in our work:

1. In RCDSU, we calculate feature statistics (mean and standard deviation) with robust statistics to weaken the influence of outliers, making the statistics close to the real values and improving the robustness of deep learning models.
2. In RCDSU and FeatureDA, we control the coefficient of variance to preserve semantics and increase shift range from the perspective of brain-inspired computer vision.
3. We combine style transfer and content transfer (RCDSU + FeatureDA) by performing style transfer on the feature map, and then performing content transfer on the feature vector learned by the feature extraction network. We perform both style transfer and content transfer with implicit robot automation when generating augmented images from original images.

## 2 Related work

### 2.1 Data augmentation

Data augmentation is a method that uses a small amount of data to generate more similar synthetic data by prior knowledge to expand the training dataset. It is an effective way to improve generalization ability and alleviate model overfitting. In image recognition tasks, to enhance the geometric invariance of convolutional networks, augmentation methods such as rotation, mirroring and random flipping are often used (Simonyan and Zisserman, 2014; Srivastava et al., 2015; He et al., 2016; Huang et al., 2019). Discarding some information in training images is also an effective way to enhance training data. Random erasing (Zhong et al., 2020) and cutout (DeVries and Taylor, 2017) crop out random rectangular regions of the input image to execute augmentation. Furthermore, there are some studies on automatic data augmentation techniques. AutoAugment (Cubuk et al., 2018) uses reinforcement learning to search for a better augmentation policy among a large number of candidates. Besides, recent studies have shown that the transformations which preserve the class identity can also be seen as effective semantic data augmentation techniques (Jaderberg et al., 2015; Bousmalis et al., 2016; Antoniou et al., 2017; Ratner et al., 2017).

### 2.2 Uncertainty modeling

Some previous work on deep learning with uncertainty (Gal and Ghahramani, 2015, 2016; Kendall and Gal, 2017) also assumes that the deep features or predictions of each sample follow a Gaussian distribution. In face recognition and person re-identification, probabilistic representations are used to resolve the problems of ambiguous faces (Shi and Jain, 2020; Amaya and Von Arnim, 2023) and data outliers/label noise (Yu et al., 2020). To simultaneously learn feature embeddings and their uncertainty, data uncertainty is applied where the uncertainty is learned via a learnable subnetwork to indicate the quality of the image (Chang et al., 2020; Shi and Jain, 2020).

### 2.3 Robust statistics

The motivation of using robust statistics is to relieve the impact of outliers, which refer to values that are far from the true data. The appearance of outliers will lead to great deviation in statistical results. Robust statistics seek to provide methods that emulate popular statistical methods, but are not excessively affected by outliers or other small departures from model assumptions (Maronna et al., 2019). Robust statistics can be utilized to detect the outliers by searching for the model fitted by the majority of the data (Rousseeuw and Hubert, 2011; Feldotto et al., 2022). There are efficient robust estimators for a series of complex problems, including covariance estimation (Cheng et al., 2019; Diakonikolas et al., 2019a), sparse estimation tasks (Balakrishnan et al., 2017; Diakonikolas et al., 2019c; Cheng et al., 2022), learning graphical models (Cheng et al., 2018; Diakonikolas et al., 2021), linear regression (Klivans et al., 2018; Diakonikolas et al., 2019d; Pensia et al., 2020), stochastic optimization (Diakonikolas et al., 2019b; DeWolf et al., 2020; Prasad et al., 2020), etc. In RCDSU, we use the property that the median is highly resistant to outliers to enhance the robustness of the model.

### 2.4 Brain-inspired computer vision

Brain-inspired methods are approaches that draw inspiration from the functionality and structure of the human brain and apply these mechanisms and principles to artificial intelligence and computer science (Zendrikov et al., 2023). Data augmentation is an important task in the field of computer vision, aiming to generate more similar synthetic data by prior knowledge to expand the training dataset. When applying brain-inspired methods to data augmentation tasks, we can explore and utilize the structure and functionality of the human brain from multiple perspectives to improve the performance of data augmentation. Designing neural network architectures inspired by brain is an important aspect. We can gain valuable insights from the visual processing mechanisms in the human brain and build neural network models with similar structures and connectivity patterns to mimic the processing and transmission of visual information (Qiu et al., 2023). We can design hierarchical neural networks where each module corresponds to different visual processing phases in the human brain (Cheng et al., 2023). For example, when we observe an image, we will pay attention to its content and style, such as a dog with painting style, a cat with sketch style, a car with photo style and so on. Therefore, we can perform style transfer and content transfer sequentially in data augmentation tasks. From the perspective of brain inspiration, the distribution of sample data can be regarded as a “spherical space”, which can be regarded as a circle in two-dimensional space and a sphere in three-dimensional space (Jeon et al., 2022). Therefore, the data augmentation strategy of each point can be determined by its position in the data distribution. Brain-inspired methods can draw inspiration from the collaborative work of multiple brain regions in the human brain, combining and analyzing data from different vision aspects (such as style and content) to improve the diversity and performance of data augmentation.

## 3 Method

### 3.1 Preliminaries

In the field of data augmentation, we have the following general formula:


(1) $$\begin{array}{l} \tilde{x}=f\left(x\right), \end{array}$$

where *f* denotes any transformation in the image space or in the feature space, *x* denotes the original image in the image space or the original feature in the feature space, and $\tilde{x}$ denotes the augmented image or feature in the corresponding space.

In this paper, *f* represents DSU, RCDSU or FeatureDA transformation. In DSU and RCDSU transformations, *x* denotes the encoded features in the intermediate layers of the network, that is, the feature maps. In the FeatureDA transformation, *x* denotes the deep features learned by a special network, that is, the feature vectors.

DSU calculates the feature mean and standard deviation by channel for each feature map, that is, calculates the feature mean and standard deviation for all pixel values of each channel. Then it calculates the variance of all feature statistics in a mini-batch, and uses the variance to generate random shifts to add to the original feature statistics. All feature statistics in a mini-batch share the same variance. More details about DSU can refer to Li et al. (2022).

### 3.2 Robust statistics for DSU

**Figure 1:**
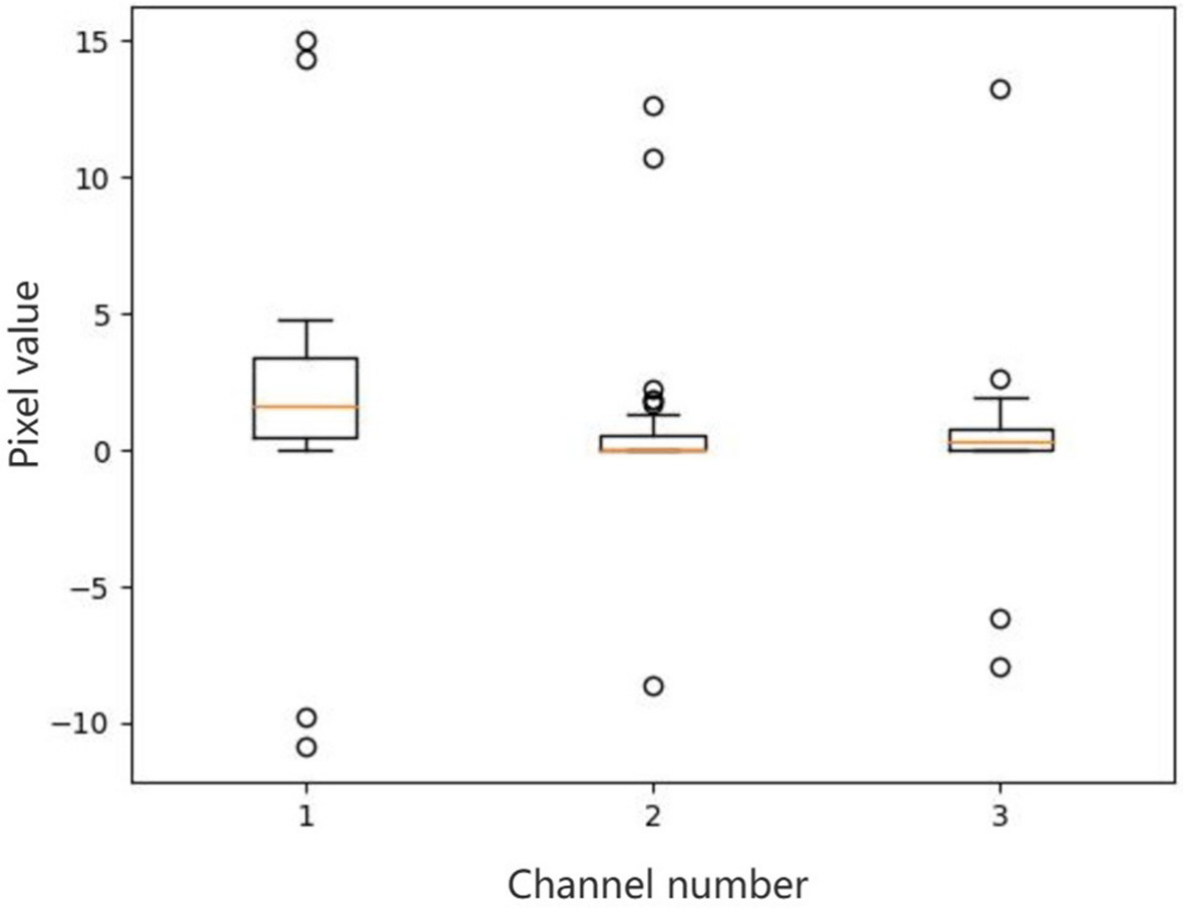
Box plots of all the pixel values of the selected channels in a feature map. There are outliers in some channels of a feature map. We select three channels which have outliers from a feature map, and then make box plots for all pixel values of each channel. For outliers, if not dealt with, they will affect the final mean and variance.

There are outliers in some channels of a feature map. We select three channels which have outliers from a feature map, and then make box plots for all pixel values of each channel. The results are shown in Figure 1. For outliers, if not dealt with, they will affect the final mean and variance.

Therefore, when calculating the mean and variance by channel, in order to alleviate the impact of outliers on the mean and variance, a method of robust statistics is used. First arrange all the pixel values of each channel from small to large. Then divide all pixel values in a channel equally into *S* segments, and the number of pixels in each segment is *HW*/*S*. Find the median *m* of all pixel values in each segment. Then calculate the average of all medians in a channel as the mean of all pixel values and calculate the variance of all medians in a channel as the variance of all pixel values.

Given *x* ∈ ℝ^*B*×*C*×*H*×*W*^ to be the features which are encoded in the intermediate layers of the network, we divide all pixels in a channel into *S* segments and denote *m* ∈ ℝ^*B*×*C*×*S*^ as the median of each segment. The feature mean μ ∈ ℝ^*B*×*C*^ and standard deviation σ ∈ ℝ^*B*×*C*^ using robust statistics can be formulated as:


(2) $$\begin{array}{l} \mu \left(x\right)=\frac{1}{S}\overset{S}{\underset{s=1}{\sum }}m_{b,c,s}, \end{array}$$

(3) $$\begin{array}{l} \sigma^{2}\left(x\right)=\frac{1}{S}\overset{S}{\underset{s=1}{\sum }}{\left(m_{b,c,s}-\mu \left(x\right)\right)}^{2}, \end{array}$$

where *b* represents the *b*th instance in a mini-batch, *c* represents the *c*th channel in a feature map, *s* represents the *s*th segment in a channel.

**Figure 2:**
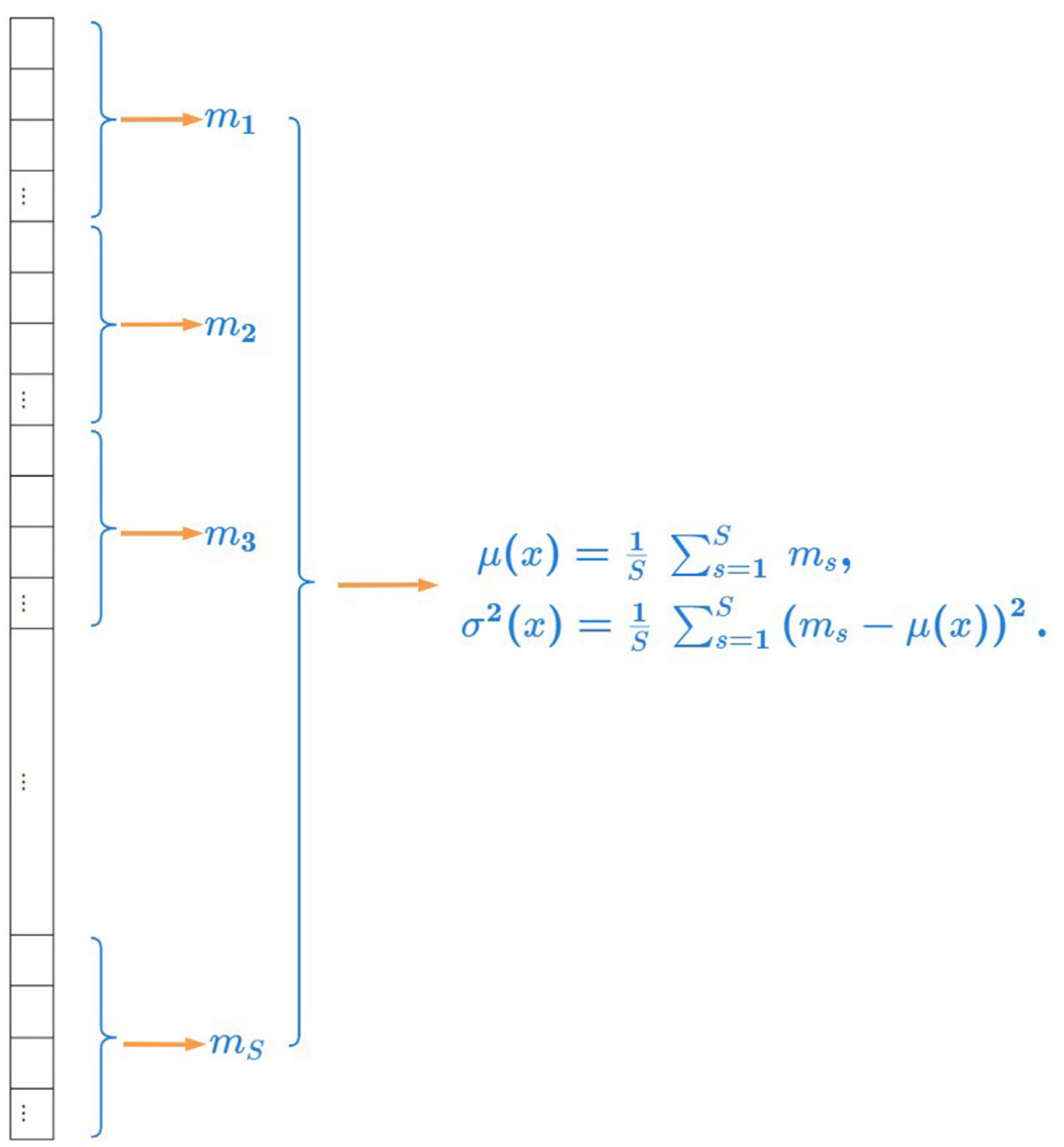
Illustration of robust statistics. We calculate the average of all medians in a channel as the mean of all pixel values and calculate the variance of all medians in a channel as the variance of all pixel values.

The illustration of robust statistics is shown in Figure 2. We calculate the average of all medians in a channel as the mean of all pixel values and calculate the variance of all medians in a channel as the variance of all pixel values.

Following DSU, we can calculate the variance of the feature statistics as follows:


(4) $$\begin{array}{l} \Sigma_{\mu }^{2}\left(x\right)=\frac{1}{B}\overset{B}{\underset{b=1}{\sum }}{\left(\mu \left(x\right)-{𝔼}_{b}\left[\mu \left(x\right)\right]\right)}^{2}, \end{array}$$

(5) $$\begin{array}{l} \Sigma_{\sigma }^{2}\left(x\right)=\frac{1}{B}\overset{B}{\underset{b=1}{\sum }}{\left(\sigma \left(x\right)-{𝔼}_{b}\left[\sigma \left(x\right)\right]\right)}^{2}, \end{array}$$

where $\Sigma_{\mu }\in {\mathbb{R}}^{C}$ and $\Sigma_{\sigma }\in {\mathbb{R}}^{C}$ represent the shift range of the feature mean μ and feature standard deviation σ, respectively.

### 3.3 Control the coefficient of variance for DSU

In the above, we calculate feature statistics with robust statistics for DSU to weaken the influence of outliers. Next we will control the coefficient of variance for DSU to make the feature statistics shift with semantic preservation and increase shift range.

According to the sphere distribution of the feature statistics, the closer to the outer layer of the sphere distribution the data point is, we hope that its shift will be smaller to avoid the semantic change of the feature statistic caused by the shift out of the boundary. And the closer to the center of the sphere distribution the data point is, we hope that its shift can be slightly larger to improve the coverage of the augmented feature statistics, increase the diversity of the augmented feature statistics and further enhance the generalization ability of the model. In order to achieve this goal, the size of the shift is controlled by multiplying a coefficient in front of the variance. We assign the coefficient of variance to each feature statistic by its Euclidean distance from the center vector. The larger the distance from the center vector is, the smaller the coefficient of variance corresponding to the feature statistic is, that is, the smaller the shift of the data point is. The smaller the distance from the center vector is, the larger the coefficient of variance corresponding to the feature statistic is, that is, the larger the shift of the data point is.

Given $\mu_{i}\in {\mathbb{R}}^{C}$ and $\sigma_{i}\in {\mathbb{R}}^{C}$ to be the feature mean and standard deviation of the *i*th instance in a mini-batch, respectively, we denote $ct_{\mu }\in {\mathbb{R}}^{C}$ and $ct_{\sigma }\in {\mathbb{R}}^{C}$ as the center of the feature statistics, which can be formulated as:


(6) $$\begin{array}{l} ct_{\mu }=\frac{1}{B}\overset{B}{\underset{i=1}{\sum }}\mu_{i}, \end{array}$$

(7) $$\begin{array}{l} ct_{\sigma }=\frac{1}{B}\overset{B}{\underset{i=1}{\sum }}\sigma_{i}. \end{array}$$

We denote *d*_μ_*i*__ as the Euclidean distance between μ_*i*_ and *ct*_μ_, and denote *d*_σ_*i*__ as the Euclidean distance between σ_*i*_ and *ct*_σ_, which can be formulated as:


(8) $$\begin{array}{l} d_{\mu_{i}}=||\mu_{i}-ct_{\mu }{||}_{2}, \end{array}$$

(9) $$\begin{array}{l} d_{\sigma_{i}}=||\sigma_{i}-ct_{\sigma }{||}_{2}. \end{array}$$

Then sort all the distances of $d_{\mu }\in {\mathbb{R}}^{B}$ and $d_{\sigma }\in {\mathbb{R}}^{B}$ in descending order respectively, and we can get the sorted distance lists, *sorted*_*distance*_μ_ and *sorted*_*distance*_σ_.

We utilize *n*_μ_*i*__ to indicate the corresponding position index of *d*_μ_*i*__ in *sorted*_*distance*_μ_ and utilize *n*_σ_*i*__ to indicate the corresponding position index of *d*_σ_*i*__ in *sorted*_*distance*_σ_, where the position index ranges from 1 to *B*.

Then the coefficients of variance are given by:


(10) $$\begin{array}{l} \lambda_{\mu_{i}}=start+\left(n_{\mu_{i}}-1\right)\left(end-start\right)/\left(B-1\right), \end{array}$$

(11) $$\begin{array}{l} \lambda_{\sigma_{i}}=start+\left(n_{\sigma_{i}}-1\right)\left(end-start\right)/\left(B-1\right), \end{array}$$

where *start* and *end* are the values set manually, and *B* represents the size of a mini-batch. *start* is the minimum value among all variance coefficients, while *end* is the maximum value.

We set λ_μ_*i*__ and λ_σ_*i*__ as the coefficient of variance to control the degree of shift. Then we obtain the augmented feature statistics:


(12) $$\begin{array}{l} {\tilde{\mu }}_{i}=\mu_{i}+X,\;X\sim N\left(0,\lambda_{\mu_{i}}\Sigma_{\mu }^{2}\right), \end{array}$$

(13) $$\begin{array}{l} {\tilde{\sigma }}_{i}=\sigma_{i}+Y,\;Y\sim N\left(0,\lambda_{\sigma_{i}}\Sigma_{\sigma }^{2}\right), \end{array}$$

where *X* and *Y* is a zero-mean multi-variate normal distribution, respectively.

The augmented feature statistics, mean $\tilde{\mu }\left(x\right)\sim N\left(\mu ,\lambda_{\mu }\Sigma_{\mu }^{2}\right)$ and standard deviation $\tilde{\sigma }\left(x\right)\sim N\left(\sigma ,\lambda_{\sigma }\Sigma_{\sigma }^{2}\right)$, can be randomly drawn from the corresponding distributions as:


(14) $$\begin{array}{l} \tilde{\mu }\left(x\right)=\mu \left(x\right)+\epsilon_{\mu }\sqrt{\lambda_{\mu }}\Sigma_{\mu }\left(x\right),\;\epsilon_{\mu }\sim N\left(0,1\right), \end{array}$$

(15) $$\begin{array}{l} \tilde{\sigma }\left(x\right)=\sigma \left(x\right)+\epsilon_{\sigma }\sqrt{\lambda_{\sigma }}\Sigma_{\sigma }\left(x\right),\;\epsilon_{\sigma }\sim N\left(0,1\right). \end{array}$$

The final formula of RCDSU is as follows:


(16) $$\begin{array}{r} \begin{array}{l} \mathrm{RCDSU}\left(x\right)=\underset{\tilde{\sigma }\left(x\right)}{\underset{︸}{\left(\sigma \left(x\right)+\epsilon_{\sigma }\sqrt{\lambda_{\sigma }}\Sigma_{\sigma }\left(x\right)\right)}}\left(\frac{x-\mu \left(x\right)}{\sigma \left(x\right)}\right)\\ +\underset{\tilde{\mu }\left(x\right)}{\underset{︸}{\left(\mu \left(x\right)+\epsilon_{\mu }\sqrt{\lambda_{\mu }}\Sigma_{\mu }\left(x\right)\right)}}, \end{array} \end{array}$$

where μ(*x*) and σ(*x*) are feature statistics calculated using the robust statistics formulas (Equations 2, 3).

**Figure 3:**
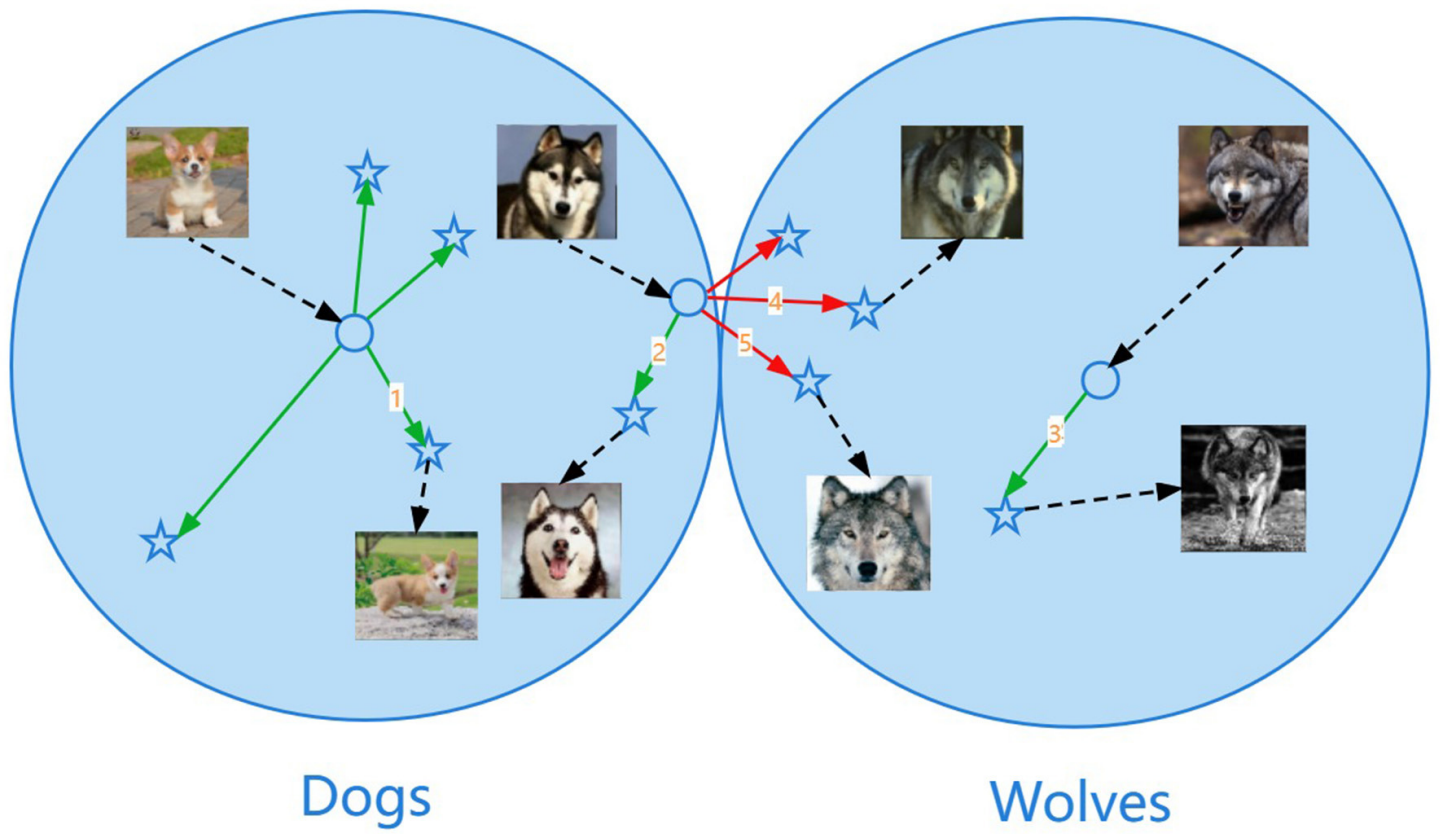
Illustration of the sphere data distribution. The data points close to the center of the sphere are not easy to break through the class boundary when shifting. For example, the shift marked as number 1 or number 3 in the figure transforms without changing the class identity and it means that the semantics are preserved. The data points close to the outermost layer of the sphere are easy to break through the class boundary when shifting, resulting in semantic changes. For example, the shift marked as number 4 or number 5 in the figure transforms from dogs to wolves and it means that the shift is too large, resulting in a change in semantics, which is the wrong shift.

The illustration of the sphere data distribution is shown in Figure 3. The data points close to the center of the sphere are not easy to break through the class boundary when shifting. For example, the shift marked as number 1 or number 3 in the figure transforms without changing the class identity and it means that the semantics are preserved. The data points close to the outermost layer of the sphere are easy to break through the class boundary when shifting, resulting in semantic changes. For example, the shift marked as number 4 or number 5 in the figure transforms from dogs to wolves and it means that the shift is too large, resulting in a change in semantics, which is the wrong shift.

### 3.4 Content transfer with FeatureDA

In the above, we introduce using Robust statistics and controlling the Coefficient of variance for DSU (RCDSU), which is utilized for style transfer. Next we will introduce Feature Data Augmentation (FeatureDA), which is utilized for content transfer. FeatureDA controls the coefficient of variance similarly to generate the augmented features with semantics unchanged and increase the coverage of augmented features.

According to the sphere distribution of the features, the closer to the outer layer of the sphere distribution the data point is, we hope that its shift will be smaller to avoid the semantic change of the feature caused by the shift out of the boundary. The closer to the center of the sphere distribution the data point is, we hope that its shift can be slightly larger to improve the coverage of the augmented features, increase the diversity of the augmented features and further improve the generalization ability of the model. In order to achieve this goal, the size of the shift is controlled by multiplying a coefficient in front of the variance. We assign the coefficient of variance to each feature by its Euclidean distance from the center vector. The larger the distance from the center vector is, the smaller the coefficient of variance corresponding to the feature is, that is, the smaller the shift of the data point is. The smaller the distance from the center vector is, the larger the coefficient of variance corresponding to the feature is, that is, the larger the shift of the data point is.

Given *a*∈ℝ^*B*×*A*^ to be the deep features and $a_{i}\in {\mathbb{R}}^{A}$ to be the deep feature of the *i*th instance in a mini-batch learned by a deep network, we denote $\Sigma_{a}^{2}$ as the variance of all features in a mini-batch, which can be formulated as:


(17) $$\begin{array}{l} \Sigma_{a}^{2}=\frac{1}{B}\overset{B}{\underset{b=1}{\sum }}{\left(a-{𝔼}_{b}\left[a\right]\right)}^{2}. \end{array}$$

We denote $ct_{a}\in {\mathbb{R}}^{A}$ as the center of the features, which can be formulated as:


(18) $$\begin{array}{l} ct_{a}=\frac{1}{B}\overset{B}{\underset{i=1}{\sum }}a_{i}. \end{array}$$

We set *d*_*a*_*i*__ as the Euclidean distance between *a*_*i*_ and *ct*_*a*_, which can be formulated as:


(19) $$\begin{array}{l} d_{a_{i}}=||a_{i}-ct_{a}|{|}_{2}. \end{array}$$

Then sort all the distances of $d_{a}\in {\mathbb{R}}^{B}$ in descending order, and we can get the sorted distance list, *sorted*_*distance*_*a*_.

We utilize *n*_*a*_*i*__ to indicate the corresponding position index of *d*_*a*_*i*__ in *sorted*_*distance*_*a*_, where the position index ranges from 1 to *B*.

Then the coefficient of variance is given by:


(20) $$\begin{array}{l} \lambda_{a_{i}}=start+\left(n_{a_{i}}-1\right)\left(end-start\right)/\left(B-1\right), \end{array}$$

where *start* and *end* are the values set manually, and *B* represents the size of a mini-batch. *start* is the minimum value among all variance coefficients, while *end* is the maximum value.

We set λ_*a*_*i*__ as the coefficient of variance to control the degree of shift. Then we obtain the augmented feature:


(21) $$\begin{array}{ll} {ã}_{i} & =\mathrm{FeatureDA}\left(a_{i}\right) \end{array}$$

(22) $$\begin{array}{l} =a_{i}+Z,\;Z\sim N\left(0,\lambda_{a_{i}}\Sigma_{a}^{2}\right), \end{array}$$

where *Z* denotes a zero-mean multi-variate normal distribution.

Finally, we can obtain the augmented feature ${ã}_{i}\;\sim \;N\left(a_{i},\lambda_{a_{i}}\Sigma_{a}^{2}\right)$.

### 3.5 Network architecture

**Figure 4:**
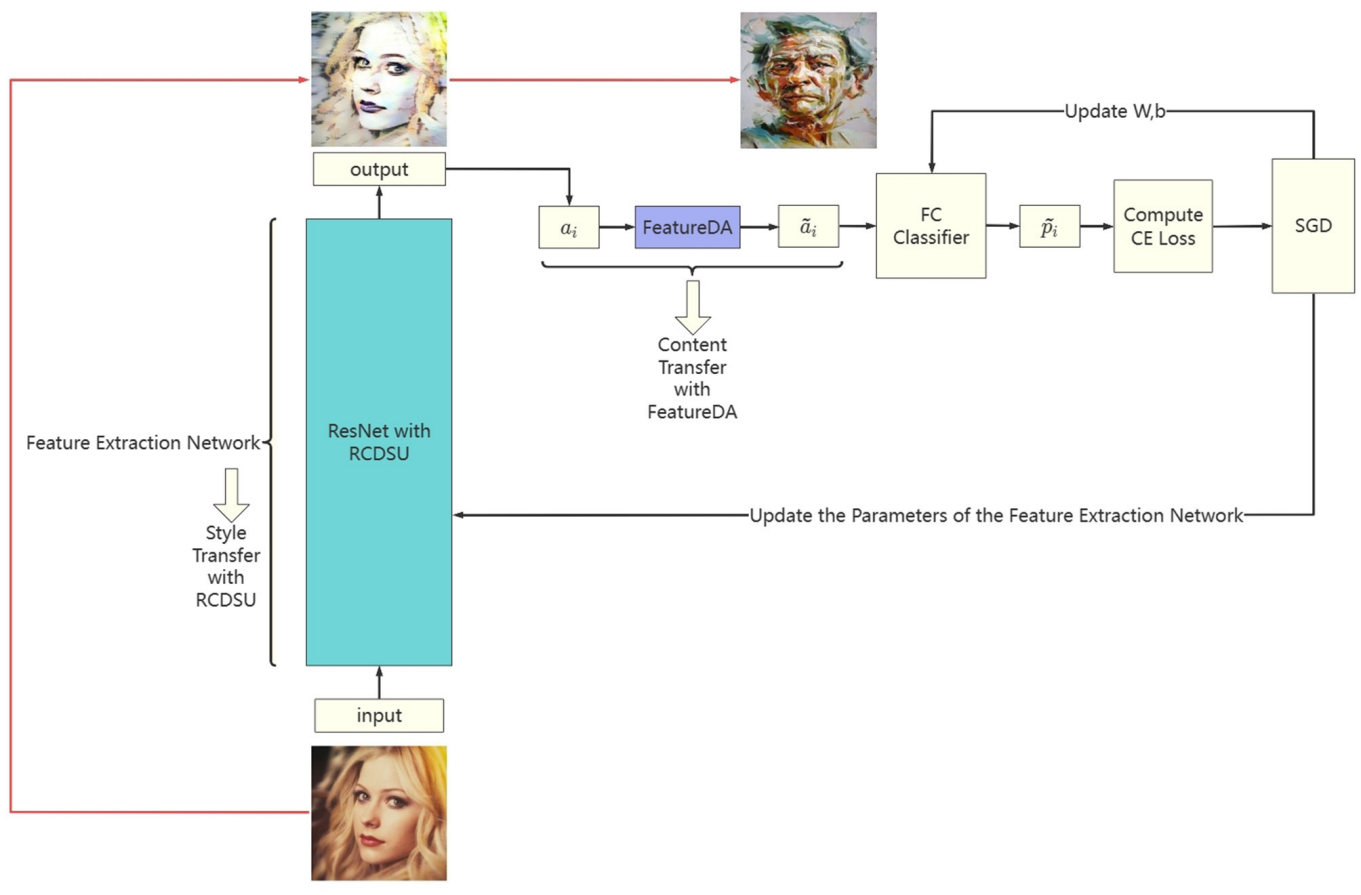
The network architecture of our method (RCDSU + FeatureDA). Note these images are for visualization only, rather than feeding into the network for training.

The network architecture of our method (RCDSU + FeatureDA) is shown in Figure 4. We use ResNet18 as the backbone. RCDSU and FeatureDA can be plug-and-play modules to be readily inserted into the network. In ResNet18, we insert RCDSU after first Conv, Max Pooling layer, 1, 2, 3, 4-th ConvBlock. After the feature extraction network, we can get the deep feature *a*_*i*_. And we can get the augmented feature ã_*i*_ by using FeatureDA. The predicted value ${\tilde{p}}_{i}$ of the augmented feature is obtained through a fully connected layer classifier. Then calculate the cross-entropy loss between the predicted value and the real value. With the stochastic gradient descent (SGD) algorithm, we can update the parameters of the feature extraction network, and update the weight matrix *W* and biases *b* of the fully connected layer. We present the pseudo code of the proposed method (RCDSU + FeatureDA) in Algorithm 1.

**Algorithm 1:** The algorithm of the proposed method.

**Input**: Intermediate feature *x*∈ℝ^*B*×*C*×*H*×*W*^
1 Compute the feature statistics μ, σ with robust statistics.
2 Compute $\Sigma_{\mu }^{2}$, $\Sigma_{\sigma }^{2}$.
3 Compute the coefficient of variance λ_μ_ and λ_σ_.
4 Style transfer with RCDSU: $\tilde{x}=\mathrm{RCDSU}\left(x\right)$.
5 Get the features *a*∈ℝ^*B*×*A*^ after the deep network.
6 Compute $\Sigma_{a}^{2}$.
7 Compute the coefficient of variance λ_*a*_*i*__.
8 Content transfer with FeatureDA: ã_*i*_ = FeatureDA(*a*_*i*_). **Output**: The augmented features ã∈ℝ^*B*×*A*^

## 4 Experiments

In this section, we empirically validate the proposed method on several tasks. First, PACS multi-style classification task is performed using our method (RCDSU + FeatureDA). We compare our method with the previously proposed methods such as pAdaIN (Nuriel et al., 2021) and MixStyle (Zhou et al., 2021). Second, FeatureDA is used alone to perform CIFAR-100 image classification task. We report the accuracy of several modern deep networks with and without FeatureDA. Third, we add Gaussian noise to PACS training data, and compare our method (RCDSU + FeatureDA) with DSU (Li et al., 2022), ISDA (Wang et al., 2021), MixStyle (Zhou et al., 2021), pAdaIN (Nuriel et al., 2021), PCL (Yao et al., 2022), SWAD (Cha et al., 2021), and MODE (Dai R. et al., 2023) to verify the robustness of our method. Fourth, we perform ablation studies of the proposed method on PACS and CIFAR-100 with models trained on ResNet.

### 4.1 Multi-style image classification

#### 4.1.1 Setup and implementation details

We choose the PACS dataset, a commonly used benchmark for multi-style image classification. PACS consists of four styles, i.e., Art Painting, Cartoon, Photo, and Sketch, with totally 9,991 images of seven classes. For evaluation, a model is trained on three styles and tested on the remaining one. Following prior work, we use ResNet18 and ResNet50 as the backbones. We compare our method (RCDSU + FeatureDA) with the previously proposed methods such as pAdaIN (Nuriel et al., 2021) and MixStyle (Zhou et al., 2021).

#### 4.1.2 Results

**Figure 5:**
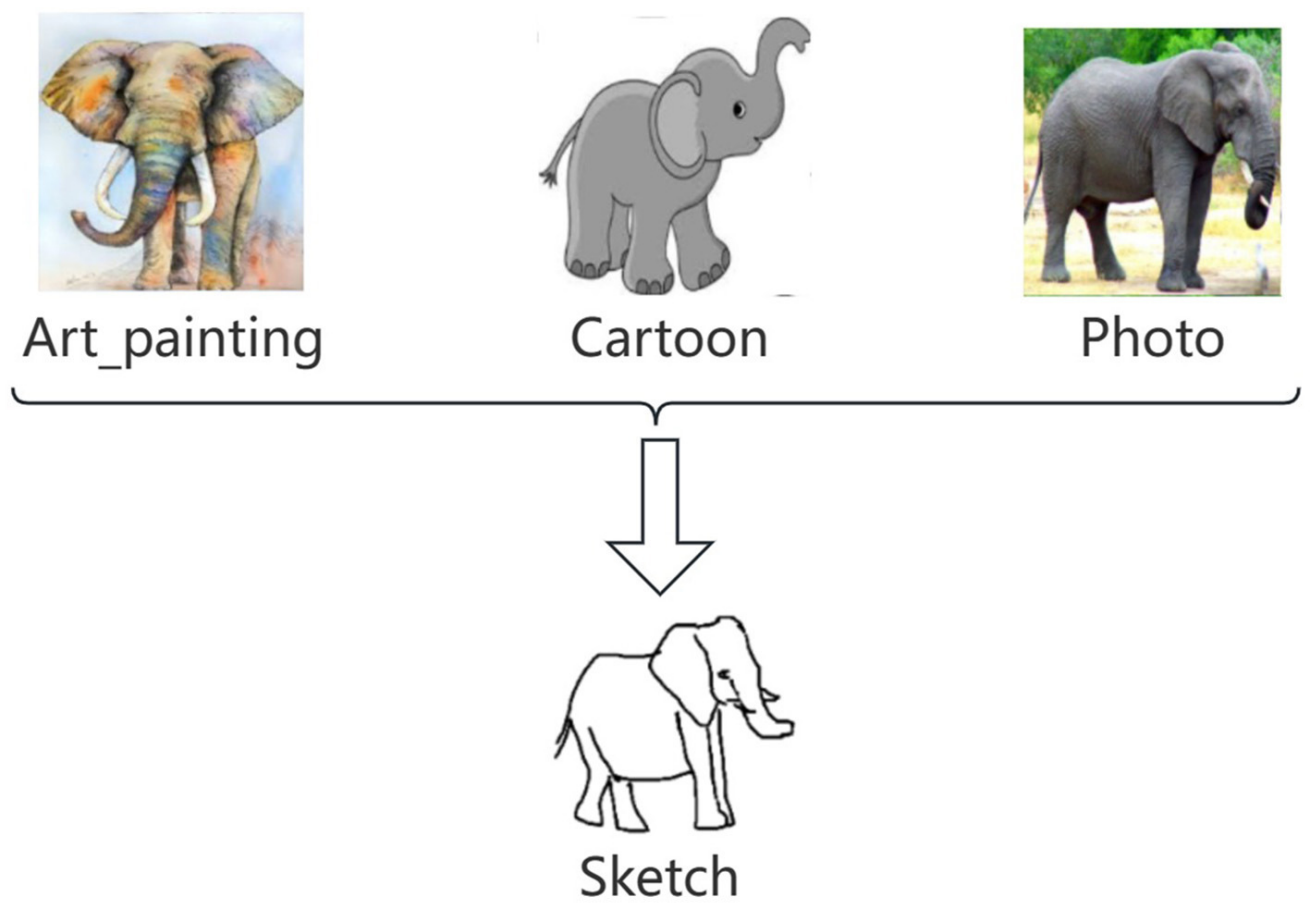
Illustration of the experiments in Sketch. We train on Art Painting, Cartoon and Photo, and test on Sketch. We can see that the style difference between training data and testing data is very large and this makes our method work well.

The experiment results, shown in Table 1, demonstrate our improvement over the baseline method on both ResNet18 and ResNet50, which shows our superiority to the conventional approach. We use Ours to denote our method (RCDSU + FeatureDA). In the last column of the table, our method improves the accuracy by an average of 1.24% compared to the previous methods, and the classification performance in Sketch is higher than other methods by over 3%. This is because our method works better when the style difference between training data and testing data is larger and the experiments in Sketch fit this very well, as shown in Figure 5. In Cartoon, our method also shows slightly better performance than previous methods. The performance in Photo is not very good because the style differences between Art Painting, Cartoon, and Photo are not very large.

**Table 1:** Experiment results of PACS multi-style classification task.

**Method**	**Art**	**Cartoon**	**Photo**	**Sketch**	**Average (%)**
Baseline	74.30	76.70	96.40	68.70	79.02
L2A-OT (Zhou et al., 2020)	83.30	78.20	96.20	73.60	82.82
pAdaIN (Nuriel et al., 2021)	81.74	76.91	96.29	75.13	82.51
MixStyle (Zhou et al., 2021)	82.30	79.00	96.30	73.80	82.85
Ours	82.72	**79.14**	94.58	**78.28**	**83.68**
Baseline	86.20	78.70	97.66	70.63	83.29
pAdaIN (Nuriel et al., 2021)	85.82	81.06	97.17	77.37	85.36
MixStyle (Zhou et al., 2021)	86.80	79.00	96.60	78.50	85.22
RSC (Huang et al., 2020)	85.40	79.70	97.60	78.20	85.22
Ours	86.68	**81.28**	97.15	**82.12**	**86.80**

Lines 1 to 5 represent the experimental results of ResNet18, and lines 6 to 10 represent the experimental results of ResNet50. The best results are bold-faced.

Our method keeps the performance of the model at a relatively high level although the accuracy of our method is not as good as that of DSU and the latest methods. RCDSU plus FeatureDA improves the robustness of the model, which can be seen in Section 4.3. We provide a novel idea for multi-style image data augmentation, that is, to improve the generalization performance of the model at the style and content level respectively.

### 4.2 FeatureDA for CIFAR-100 image classification

#### 4.2.1 Setup and implementation details

The CIFAR-100 dataset consists of 32 × 32 colored natural images in 100 classes, with 50,000 images for training and 10,000 images for testing. Since CIFAR-100 belongs to a single-style dataset, that is, there are not great style differences between training data and testing data. Therefore, style transfer is not required for data augmentation and only content transfer is required. We use FeatureDA alone to perform the CIFAR-100 image classification task.

#### 4.2.2 Results

We report the accuracy of several modern deep networks with and without FeatureDA on CIFAR-100 in Table 2. On the single-style dataset CIFAR-100, FeatureDA can improve the classification accuracy of the model by an average of 0.92%, and is applicable to a variety of networks. It proves that FeatureDA can indeed be used as an efficient data augmentation method based on content transfer to improve the generalization ability of the model at the content level.

**Table 2:** Evaluation (%) of FeatureDA on CIFAR-100 with different models.

**Networks**	**CIFAR-100**		
	**Basic**	**FeatureDA**	**Improvement**
ResNet-32	68.80	70.04	1.24
ResNet-110	71.33	74.19	2.86
SE-ResNet-110	72.70	74.04	1.34
Wide-ResNet-16-8	79.76	79.98	0.22
Wide-ResNet-28-10	81.47	81.91	0.44
ResNeXt-29, 8x64d	81.84	82.44	0.60
DenseNet-BC-100-12	77.39	77.81	0.42
Shake-Shake (26, 2x32d)	79.88	80.46	0.58
Shake-Shake (26, 2x112d)	82.58	83.13	0.55
Average	–	–	0.92

### 4.3 Robustness to noise

#### 4.3.1 Setup and implementation details

We add Gaussian noise that follows $N\left(0,noise\text{\_}std^{2}\right)$ to the feature map of each sample in PACS training data, and then perform the PACS multi-style classification task. *noise*_*std* is selected from {0.25, 0.5, 1, 1.5, 2}. We compare our method (RCDSU + FeatureDA) with DSU (Li et al., 2022), ISDA (Wang et al., 2021), MixStyle (Zhou et al., 2021), pAdaIN (Nuriel et al., 2021), PCL (Yao et al., 2022), SWAD (Cha et al., 2021), and MODE (Dai R. et al., 2023) to verify the robustness of our method.

#### 4.3.2 Results

**Figure 6:**
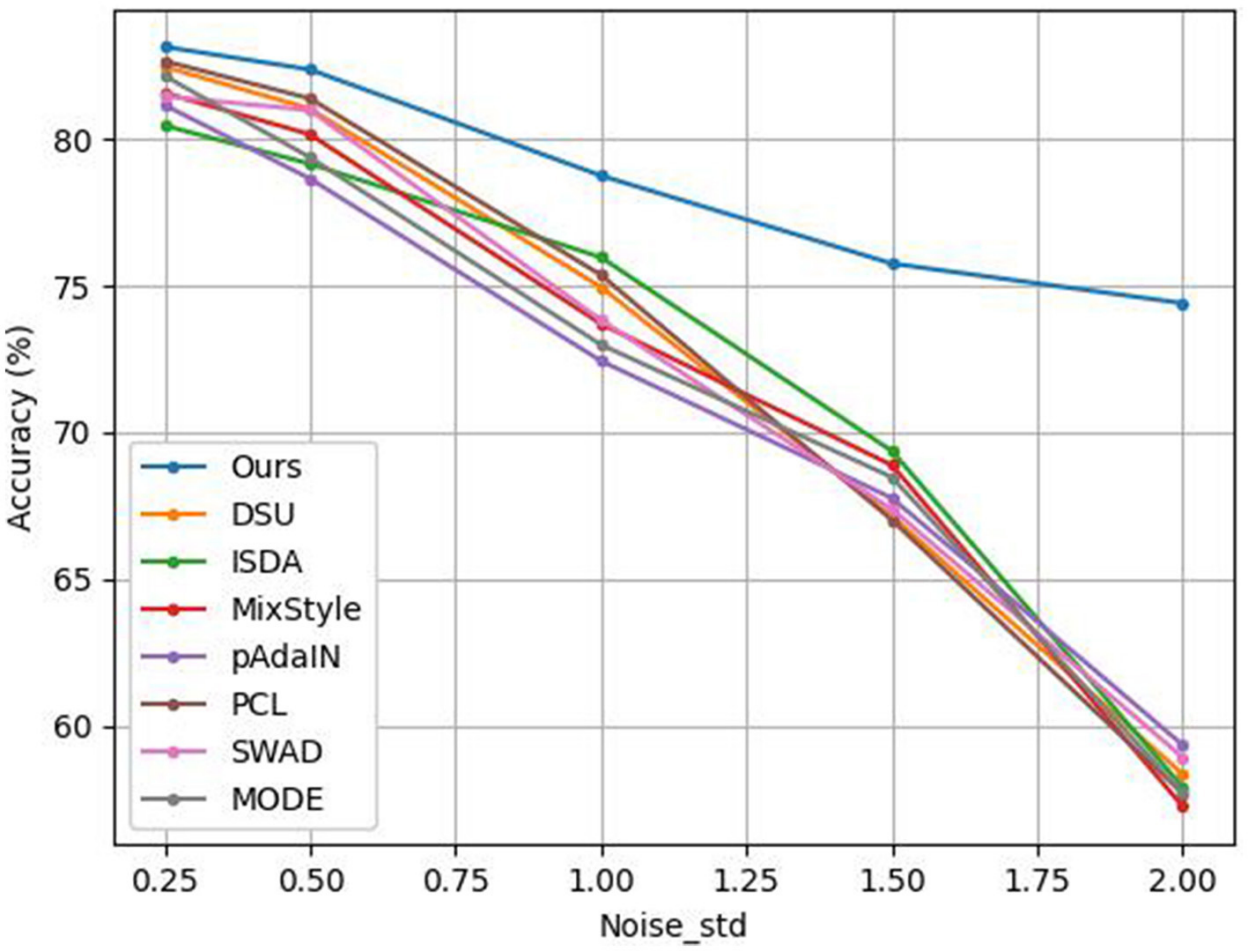
Experiment results of adding Gaussian noise to PACS training data with different methods.

The results are shown in Figure 6. It can be seen that when we add Gaussian noise that follows $N\left(0,noise\text{\_}std^{2}\right)$ to the feature map of each sample in PACS training data, the classification accuracy of our method is better than that of DSU and other methods. When *noise*_*std* is set to 2, our method outperforms other methods by over 15%. This is because our method considers outliers and uses robust statistics to weaken the influence of outliers.

MODE performs distribution exploration in an uncertainty subset that shares the same semantic factors with the training domains. However, it does not consider the outliers. So its performance degrades dramatically in the case of high noise. Besides, the mean and standard deviation in DSU, MixStyle and pAdaIN, and the covariance matrix in ISDA are affected by outliers. Outliers are not handled in these methods. Therefore, they don't perform well in high noise.

When the training data is mixed with noise, the model trained by our method can still maintain good generalization ability. It shows that our method can indeed improve the robustness and the ability to resist outlier disturbances of the model after using robust statistics. That is to say, our method is more robust than DSU and other methods. In other words, after adding a small amount of Gaussian noise to each training sample, our method can still learn the key features of each sample. However, DSU and other methods cannot learn the key features of each sample well under the disturbance of a small amount of Gaussian noise. That is to say, when the training data is mixed with noise, our method can make the deep network perform feature extraction better, compared to DSU and other methods.

### 4.4 Ablation study

Next we will perform ablation studies of the proposed method on PACS and CIFAR-100 with models trained on ResNet. We will conduct the following ablation studies respectively: (1) Set different starting and ending points when FeatureDA controls the variance coefficient. (2) Set different starting and ending points when RCDSU controls the variance coefficient. (3) Set the number of segments when RCDSU uses robust statistics. (4) Conduct a series of experiments on the combinations of RCDSU and FeatureDA.

We use FeatureDA(no coefficient) to represent FeatureDA without controlling the coefficient of variance, and use RCDSU(no modules) to represent RCDSU that neither uses robust statistics nor controls the coefficient of variance.

#### 4.4.1 Controlling the variance coefficient in FeatureDA

We set different starting and ending points, *start* and *end*, when FeatureDA controls the variance coefficient.

##### 4.4.1.1 CIFAR-100 image classification task

As shown in Table 3, when we use FeatureDA to perform the CIFAR-100 image classification task with Resnet-32, setting *start* and *end* to 0.4 and 0.9 works best.

**Table 3:** Setting different starting and ending points when FeatureDA controls the variance coefficient on CIFAR-100 image classification task.

**Networks**	**Start, end**	**CIFAR-100**
ResNet-32	FeatureDA (no coefficient)	68.71
	FeatureDA (start = 0.5, end = 2)	67.84
	FeatureDA (start = 0.8, end = 1.5)	68.45
	FeatureDA (start = 0.8, end = 1.2)	68.57
	FeatureDA (start = 0.8, end = 1.0)	68.59
	FeatureDA (start = 0.5, end = 1.0)	69.50
	FeatureDA (start = 0.5, end = 0.8)	68.99
	FeatureDA (start = 0.4, end = 0.9)	**70.04**
	FeatureDA (start = 0.3, end = 0.9)	69.17
	FeatureDA (start = 0.3, end = 0.8)	69.51

Bold values indicate the best result.

In the CIFAR-100 dataset, the difference between all features in a mini-batch is too large, that is, the variance is too large. This means that the shift will be large and the semantics will change. So the coefficient multiplied in front of the variance should be < 1 to make the variance smaller. We can see that setting *start* and *end* to 0.4 and 0.9 works better than setting *start* and *end* to 0.5 and 2 because the coefficients of the former are smaller. We reduce the shift by making the coefficient small to avoid semantic changes.

However, the coefficient cannot be infinitely small. As the shift gets smaller, the diversity of augmented features will decrease. We can see that setting *start* and *end* to 0.4 and 0.9 works better than setting *start* and *end* to 0.3 and 0.8 because the diversity of the latter are smaller. The coefficients can be neither too large nor too small. We need to find a balance between not changing the semantics and keeping the diversity of augmented features not too small.

Both ISDA and FeatureDA essentially add a random vector following a zero-mean multi-variate normal distribution to the original feature vector, and each value of the random vector is a random quantity that fluctuates around 0. Because the random vectors of FeatureDA and ISDA both fluctuate around 0, the difference between using the variance of all features in a mini-batch and using the covariance of all features in a class is actually not that big.

##### 4.4.1.2 PACS multi-style classification task

As shown in Table 4, when we use FeatureDA to perform the PACS multi-style classification task with ResNet18, setting *start* and *end* to 2 and 2.5 works best.

**Table 4:** Setting different starting and ending points when FeatureDA controls the variance coefficient on PACS multi-style classification task.

**Start, end**	**Accuracy (%)**
FeatureDA (no coefficient)	80.5350
FeatureDA (start = 0.5, end = 2)	80.7000
FeatureDA (start = 0.8, end = 1.5)	80.7000
FeatureDA (start = 0.5, end = 1.0)	80.3225
FeatureDA (start = 0.4, end = 0.9)	80.2925
FeatureDA (start = 1, end = 2)	80.7400
FeatureDA (start = 1, end = 1.5)	80.6175
FeatureDA (start = 1.5, end = 2)	80.8025
FeatureDA (start = 1.5, end = 2.5)	80.9300
FeatureDA (start = 2, end = 2.5)	**81.1475**
FeatureDA (start = 2, end = 3)	81.0350
FeatureDA (start = 2.5, end = 3)	80.9200

Bold values indicate the best result.

In the PACS dataset, the difference between all features in a mini-batch is too small, that is, the variance is too small. This means that the shift range will be small and the diversity of augmented features will decrease. So the coefficient multiplied in front of the variance should be > 1 to make the variance larger. We can see that setting *start* and *end* to 2 and 2.5 works better than setting *start* and *end* to 0.5 and 2 because the coefficients of the former are larger. We increase the shift range by enlarging the coefficient.

However, the coefficient cannot be infinitely large. As the shift gets larger, the semantics will change. We can see that setting *start* and *end* to 2 and 2.5 works better than setting *start* and *end* to 2.5 and 3 because the semantics of the latter change. The coefficients can be neither too large nor too small. We need to find a balance between not changing the semantics and keeping the diversity of augmented features not too small.

#### 4.4.2 Controlling the variance coefficient in RCDSU

We set different starting and ending points, *start* and *end*, when RCDSU controls variance coefficient. As shown in Table 5, when we use RCDSU to perform the PACS multi-style classification task with ResNet18, setting *start* and *end* to 0.7 and 2 works best.

**Table 5:** Setting different starting and ending points when RCDSU controls variance coefficient on PACS multi-style classification task.

**Start, end**	**Accuracy (%)**
RCDSU (no modules)	83.1125
RCDSU (start = 0.5, end = 2)	83.4600
RCDSU (start = 0.8, end = 1.5)	82.9900
RCDSU (start = 0.5, end = 1)	82.8675
RCDSU (start = 1, end = 2)	82.8000
RCDSU (start = 0.8, end = 2)	83.1350
RCDSU (start = 0.5, end = 1.5)	82.9650
RCDSU (start = 0.4, end = 2)	83.2775
RCDSU (start = 0.3, end = 2)	83.1050
RCDSU (start = 0.6, end =2)	83.4175
RCDSU (start = 0.7, end = 2)	**83.5250**

Bold values indicate the best result.

In order to avoid the semantic change, we set *start* to 0.7 to reduce the shift of the data point which is close to the outer layer of the sphere distribution. In order to increase the diversity of the augmented feature statistics, we set *end* to 2 to increase the shift of the data point which is close to the center of the sphere distribution.

We can see that setting *start* and *end* to 0.7 and 2 works better than setting *start* and *end* to 0.5 and 1 because the coefficients of the former are larger and the diversity of the former is greater. We can also see that setting *start* and *end* to 0.7 and 2 works better than setting *start* and *end* to 1 and 2 because the coefficients of the latter are larger and the semantics of the latter change. The coefficients can be neither too large nor too small. We need to find a balance between not changing the semantics and keeping the diversity of augmented features not too small.

#### 4.4.3 Using robust statistics in RCDSU

**Figure 7:**
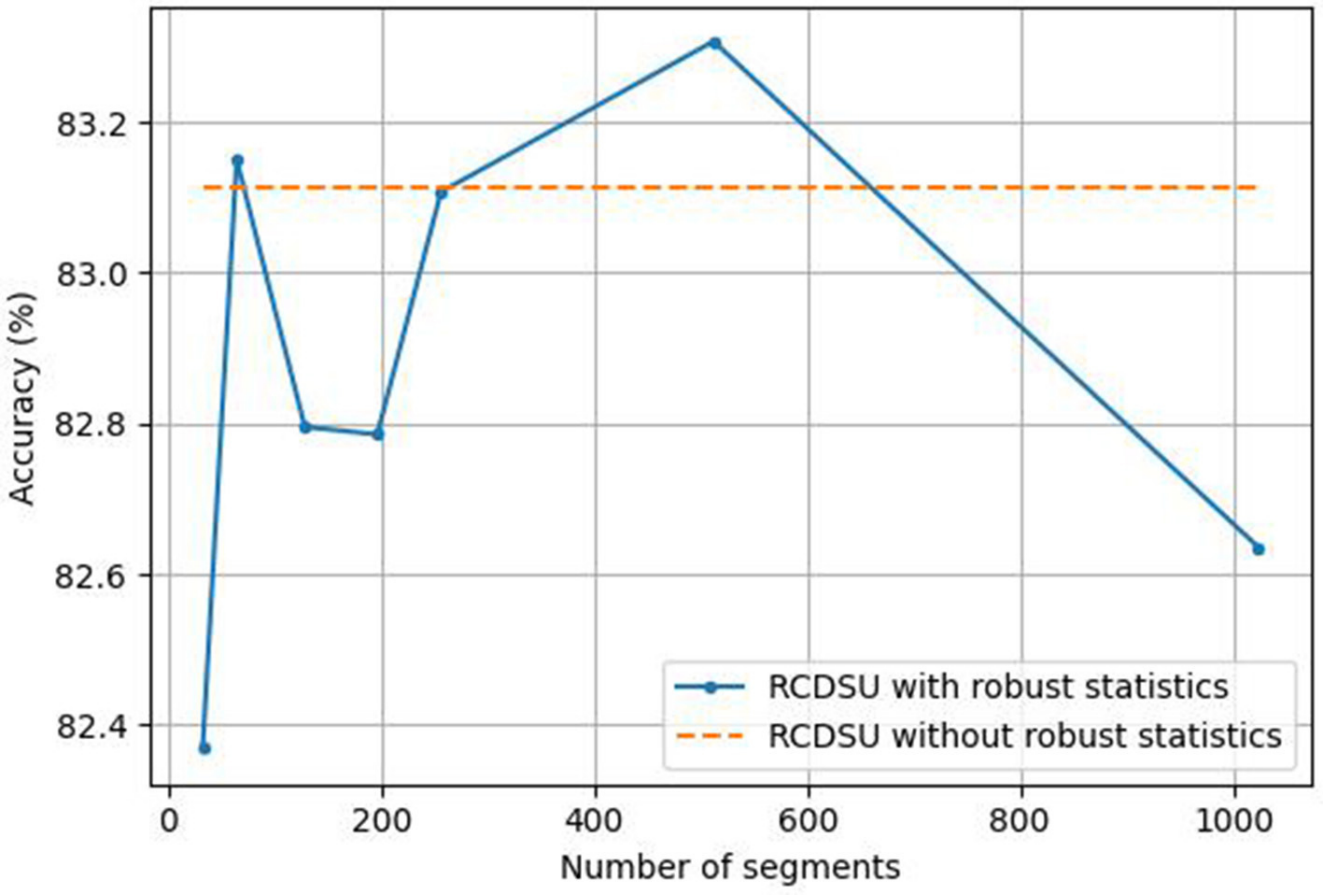
Setting the number of segments *S* for RCDSU with robust statistics.

We set the number of segments *S* to different values when RCDSU uses robust statistics. *S* is selected from {32, 64, 128, 196, 256, 512, 1, 024}. As shown in Figure 7, when we use RCDSU to perform the PACS multi-style classification task with ResNet18, setting the number of segments *S* to 512 works best.

The number of segments can be neither too large nor too small. When the number of segments is set to 32, the number of segments is too small, so that the mean and variance of all medians cannot approach the true mean and variance. When the number of segments is set to 1,024, the number of segments is too large, resulting in increased calculation costs, and at the same time, it cannot well avoid the influence of outliers. So we need to find a balance between approaching the true mean and variance and avoiding the influence of outliers.

#### 4.4.4 Combinations of RCDSU and FeatureDA

##### 4.4.4.1 PACS multi-style classification task

As shown in Table 6, when we combine RCDSU and FeatureDA to perform the PACS multi-style classification task with ResNet18, setting the number of segments *S* to 512 for RCDSU, *start* and *end* to 0.7 and 2 for RCDSU, and *start* and *end* to 2 and 2.5 for FeatureDA works best.

**Table 6:** Different combinations of RCDSU and FeatureDA on PACS multi-style classification task.

**Different combinations**	**Accuracy (%)**
RCDSU (start = 0.7, end = 2)	83.5250
RCDSU (S = 512)	83.3075
FeatureDA (start = 2, end = 2.5)	81.1475
RCDSU (start = 0.7, end = 2) + FeatureDA (start = 2, end = 2.5)	83.6100
RCDSU (S = 512) + FeatureDA (start = 2, end = 2.5)	83.4100
RCDSU (start = 0.7, end = 2, S = 512)	83.5900
RCDSU (start = 0.7, end = 2, S = 512) + FeatureDA (start = 2, end = 2.5)	**83.6800**

Bold values indicate the best result.

For the three modules, controlling the variance coefficient in FeatureDA, controlling the variance coefficient in RCDSU, and using robust statistics in RCDSU, we can see that pairwise combinations of the three modules work better than single modules. The combination of three modules works better than all combinations of two. This proves that each module in our method is effective.

##### 4.4.4.2 CIFAR-100 image classification task

As shown in Table 7, when we use RCDSU alone or use RCDSU plus FeatureDA to perform the CIFAR-100 image classification task with ResNet-32, the results are not excellent. This shows that as a style transfer module, RCDSU can not be used to perform the CIFAR-100 image classification task because CIFAR-100 is a single-style dataset and there are not large style differences between training data and testing data in CIFAR-100. RCDSU can only be used in the multi-style dataset classification task. As a content transfer module, FeatureDA improves the generalization ability of the model at the content level, which works on any dataset, so FeatureDA can be regarded as a data augmentation method that can be used in any classification task.

**Table 7:** Experiment results of combining RCDSU and FeatureDA on CIFAR-100 image classification task.

**Networks**	**Different combinations**	**CIFAR-100**
	RCDSU (no modules)	59.98
ResNet-32	FeatureDA (start = 0.4, end = 0.9)	70.04
	RCDSU (no modules) + FeatureDA (start = 0.4, end = 0.9)	51.65

## 5 Conclusion

In this paper, we proposed a brain-inspired semantic data augmentation method consisting of RCDSU and FeatureDA to perform style transfer and content transfer in the feature space. RCDSU used robust statistics to calculate feature statistics, improving the robustness of deep models. Based on the idea of spherical data distribution, we controlled the coefficient of variance for RCDSU and FeatureDA to preserve semantics and increase shift range. On PACS multi-style classification task, RCDSU plus FeatureDA achieved competitive accuracy. After adding Gaussian noise to PACS dataset, RCDSU plus FeatureDA showed strong robustness against outliers. FeatureDA achieved excellent results on CIFAR-100 image classification task. RCDSU plus FeatureDA can be applied as a novel semantic data augmentation method with implicit robot automation which is suitable for multi-style datasets. Experiment results demonstrated the effectiveness of the proposed method in improving the generalization ability of the model at the style and content level. Our augmentation method is based on the feature level. Thus, for future work, we will design a decoder to restore features to images, and generate some interesting and unexpected images. In addition, our method can be applied to situations where there are great differences between actual scenes and training scenes.

## Data availability statement

The original contributions presented in the study are included in the article/supplementary material, further inquiries can be directed to the corresponding author.

## Author contributions

WW: Conceptualization, Data curation, Writing – original draft, Writing – review & editing. ZS: Funding acquisition, Software, Supervision, Validation, Visualization, Writing – review & editing. CL: Validation, Writing – review & editing.

## References

[B1] Amaya C. Von Arnim A. (2023). Neurorobotic reinforcement learning for domains with parametrical uncertainty. Front. Neurorobot. 17:1239581. 10.3389/fnbot.2023.1239581 37965072 PMC10642204

[B2] Antoniou A. Storkey A. Edwards H. (2017). Data augmentation generative adversarial networks. arXiv [Preprint]. arXiv:1711.04340. 10.48550/arXiv.1711.04340

[B3] Balakrishnan S. Du S. S. Li J. Singh A. (2017). “Computationally efficient robust sparse estimation in high dimensions,” in Conference on Learning Theory (PMLR), 169–212.

[B4] Bousmalis K. Silberman N. Dohan D. Erhan D. Krishnan D. (2016). “Unsupervised pixel-level domain adaptation with generative adversarial networks,” in Proceedings of the IEEE Conference on Computer Vision and Pattern Recognition, 3722–3731.

[B5] Bowles C. Chen L. Guerrero R. Bentley P. Gunn R. Hammers A.. (2018). Gan augmentation: augmenting training data using generative adversarial networks. arXiv [Preprint]. arXiv:1810.10863. 10.48550/arXiv.1810.10863

[B6] Cauli N. Reforgiato Recupero D. (2022). Survey on videos data augmentation for deep learning models. Future Internet 14:93. 10.3390/fi14030093

[B7] Cha J. Chun S. Lee K. Cho H.-C. Park S. Lee Y.. (2021). “Swad: Domain Generalization by Seeking Flat Minima,” in Advances in Neural Information Processing Systems, Vol. 31, 22405–22418.

[B8] Chang J. Lan Z. Cheng C. Wei Y. (2020). “Data uncertainty learning in face recognition,” in 2020 IEEE/CVF Conference on Computer Vision and Pattern Recognition (CVPR) (Seattle, WA: IEEE). 10.1109/CVPR42600.2020.00575

[B9] Cheng Y. Diakonikolas I. Ge R. Gupta S. Kane D. Soltanolkotabi M.. (2022). “Outlier-robust Sparse Estimation via Non-convex Optimization,” in Advances in Neural Information Processing Systems, Vol. 35, 7318–7327.

[B10] Cheng Y. Diakonikolas I. Ge R. Woodruff D. P. (2019). “Faster algorithms for high-dimensional robust covariance estimation,” in Conference on Learning Theory (PMLR), 727–757. 10.1137/1.9781611975482.171

[B11] Cheng Y. Diakonikolas I. Kane D. Stewart A. (2018). “Robust learning of fixed-structure Bayesian networks,” in Advances in Neural Information Processing Systems, Vol. 31 (Montreal, QC).

[B12] Cheng Z. Wang X. Li J. (2023). Promatch: semi-supervised learning with prototype consistency. Mathematics 11:3537. 10.3390/math11163537

[B13] Cubuk E. D. Zoph B. Mane D. Vasudevan V. Le Q. V. (2018). Autoaugment: learning augmentation policies from data. arXiv [Preprint]. arXiv:1805.09501. 10.48550/arXiv.1805.09501

[B14] Dai R. Zhang Y. Fang Z. Han B. Tian X. (2023b). Moderately distributional exploration for domain generalization. arXiv [Preprint]. arXiv:2304.13976. 10.48550/arXiv.2304.13976

[B15] Dai H. Liu Z. Liao W. Huang X. Wu Z. Zhao L.. (2023a). Auggpt: leveraging chatgpt for text data augmentation. arXiv [Preprint]. arXiv:2302.13007. 10.48550/arXiv.2302.13007

[B16] DeVries T. Taylor G. W. (2017). Improved regularization of convolutional neural networks with cutout. arXiv [Preprint]. arXiv:1708.04552. 10.48550/arXiv. 708.04552

[B17] DeWolf T. Jaworski P. Eliasmith C. (2020). Nengo and low-power ai hardware for robust, embedded neurorobotics. Front. Neurorobot. 14:568359. 10.3389/fnbot.2020.568359 33162886 PMC7581863

[B18] Diakonikolas I. Kamath G. Kane D. Li J. Moitra A. Stewart A.. (2019a). Robust estimators in high-dimensions without the computational intractability. SIAM J. Comput. 48, 742–864. 10.1137/17M1126680

[B19] Diakonikolas I. Kane D. M. Stewart A. Sun Y. (2021). “Outlier-robust learning of Ising models under Dobrushin's condition,” in Conference on Learning Theory (PMLR), 1645–1682.

[B20] Diakonikolas I. Kong W. Stewart A. (2019d). “Efficient algorithms and lower bounds for robust linear regression,” in Proceedings of the Thirtieth Annual ACM-SIAM Symposium on Discrete Algorithms (Philadelphia, PA: Society for Industrial and Applied Mathematics), 2745–2754. 10.1137/1.9781611975482.170

[B21] Diakonikolas I. Kamath G. Kane D. Li J. Steinhardt J. Stewart A.. (2019b). “Sever: a robust meta-algorithm for stochastic optimization,” in International Conference on Machine Learning (PMLR), 1596–1606.

[B22] Diakonikolas I. Kane D. Karmalkar S. Price E. Stewart A. (2019c). “Outlier-robust High-dimensional Sparse Estimation via Iterative Filtering,” in Advances in Neural Information Processing Systems, Vol. 32.

[B23] Eckert D. Vesal S. Ritschl L. Kappler S. Maier A. (2020). “Deep learning-based denoising of mammographic images using physics-driven data augmentation,” in Bildverarbeitung für die Medizin 2020: Algorithmen-Systeme-Anwendungen. Proceedings des Workshops vom 15. bis 17. März 2020 in Berlin (Wiesbaden: Springer Fachmedien Wiesbaden), 94–100.

[B24] Fang T. Zhou W. Liu F. Zhang H. Song Y. Chen M. (2022). On-the-fly denoising for data augmentation in natural language understanding. arXiv [Preprint]. arXiv:2212.10558. 10.48550/arXiv.2212.10558

[B25] Feldotto B. Soare C. Knoll A. Sriya P. Astill S. de Kamps S. (2022). Evaluating muscle synergies with EMG data and physics simulation in the neurorobotics platform. Front. Neurorobot. 16:856797. 10.3389/fnbot.2022.856797 35903555 PMC9315385

[B26] Gal Y. Ghahramani Z. (2015). Bayesian convolutional neural networks with Bernoulli approximate variational inference. arXiv [Preprint]. arXiv:1506.02158. 10.48550/arXiv.1506.02158

[B27] Gal Y. Ghahramani Z. (2016). “Dropout as a Bayesian approximation: representing model uncertainty in deep learning,” in International Conference on Machine Learning (PMLR), 1050–1059.

[B28] Gorpincenko A. Mackiewicz M. (2022). “Extending temporal data augmentation for video action recognition,” in International Conference on Image and Vision Computing New Zealand (Cham: Springer Nature Switzerland), 104–118.

[B29] He K. Zhang X. Ren S. Sun J. (2016). “Deep residual learning for image recognition,” in 2016 IEEE Conference on Computer Vision and Pattern Recognition (CVPR) (Las Vegas, NV: IEEE). 10.1109/CVPR.2016.90

[B30] Huang G. Liu Z. Pleiss G. Maaten L. Weinberger K. Q. (2019). Convolutional networks with dense connectivity. IEEE Trans. Pattern Anal. Mach. Intell. 44, 8704–8716. 10.1109/TPAMI.2019.2918284 31135351

[B31] Huang Z. Wang H. Xing E. P. Huang D. (2020). “Self-challenging improves cross-domain generalization,” in Computer Vision ECCV 2020 - *16th European Conference, 2020, Proceedings, Vol. 12347*, eds Vedaldi A. Bischof H. Brox T. Frahm J.-M. (Berlin: Springer), 124–140. 10.1007/978-3-030-58536-5_8

[B32] Jaderberg M. Simonyan K. Vedaldi A. Zisserman A. (2015). Reading text in the wild with convolutional neural networks. Int. J. Comput. Vis. 116, 1–20. 10.1007/s11263-015-0823-z

[B33] Jeon H. Ko H. K. Lee S. Jo J. Seo J. (2022). “Uniform manifold approximation with two-phase optimization,” in 2022 IEEE Visualization and Visual Analytics (VIS) (Oklahoma City: IEEE), 80–84.

[B34] Kendall A. Gal Y. (2017). “What uncertainties do we need in Bayesian deep learning for computer vision?” in Advances in Neural Information Processing Systems, Vol. 30 (Long Beach, CA).

[B35] Kim T. Kim J. Shim M. Yun S. Kang M. Wee D. Lee S. (2022). Exploring temporally dynamic data augmentation for video recognition. arXiv [Preprint]. arXiv:2206.15015. 10.48550/arXiv.2206.15015

[B36] Klivans A. Kothari P. K. Meka R. (2018). “Efficient algorithms for outlier-robust regression,” in Conference On Learning Theory (PMLR), 1420–1430.

[B37] Krizhevsky A. Sutskever I. Hinton G. E. (2017). Imagenet classification with deep convolutional neural networks. Commun. ACM 60, 84–90. 10.1145/3065386

[B38] Krizhevsky A. (2009). Learning Multiple Layers of Features from Tiny Images.

[B39] Li X. Dai Y. Ge Y. Liu J. Shan Y. Duan L. Y. (2022). Uncertainty modeling for out-of-distribution generalization. arXiv [Preprint]. arXiv:2202.03958. 10.48550/arXiv.2202.03958

[B40] Liu J. Wu C.-H. Wang Y. Xu Q. Zhou Y. Huang H.. (2020). “Learning raw image denoising with bayer pattern unification and bayer preserving augmentation,” in 2019 IEEE/CVF Conference on Computer Vision and Pattern Recognition Workshops (CVPRW) (Long Beach, CA: IEEE). 10.1109/CVPRW.2019.00259

[B41] Liu Q. Li J. Wang X. Zhao W. (2023). Attentive neighborhood feature augmentation for semi-supervised learning. Intell. Autom. Soft Comput. 37, 1753–1771. 10.32604/iasc.2023.039600

[B42] Luo J. Lei W. Hou F. Wang C. Ren Q. Zhang S.. (2021). GPR B-scan image denoising via multi-scale convolutional autoencoder with data augmentation. Electronics 10:1269. 10.3390/electronics10111269

[B43] Maronna R. A. Martin R. D. Yohai V. J. Salibián-Barrera M. (2019). Robust Statistics: Theory and Methods (with R). Hoboken, NJ: John Wiley & Sons. 10.1002/9781119214656

[B44] Nuriel O. Benaim S. Wolf L. (2021). “Permuted ADaIN: reducing the bias towards global statistics in image classification,” in Proceedings of the IEEE/CVF Conference on Computer Vision and Pattern Recognition, 9482–9491.

[B45] Pensia A. Jog V. Loh P.-L. (2020). Robust regression with covariate filtering: heavy tails and adversarial contamination. arXiv [Preprint]. arXiv:2009.12976. 10.48550/arXiv.2009.12976

[B46] Prasad A. Suggala A. S. Balakrishnan S. Ravikumar P. (2020). Robust estimation via robust gradient estimation. J. R. Stat. Soc. B: Stat. Methodol. 82, 601–627. 10.1111/rssb.12364

[B47] Qiu Z. Wang X. Ma H. Hou S. Li J. Li Z.. (2023). Instance reweighting adversarial training based on confused label. Intell. Autom. Soft Comput. 37, 1243–1256. 10.32604/iasc.2023.038241

[B48] Ratner A. J. Ehrenberg H. Hussain Z. Dunnmon J. Ré C. (2017). “Learning to compose domain-specific transformations for data augmentation,” in Advances in Neural Information Processing Systems, Vol. 30 (Long Beach, CA). PMC5786274 29375240

[B49] Rousseeuw P. J. Hubert M. (2011). Robust statistics for outlier detection. Wiley Interdiscip. Rev.: Data Min. Knowl. Discov. 1, 73–79. 10.1002/widm.2

[B50] Shi Y. Jain A. (2020). “Probabilistic face embeddings,” in 2019 IEEE/CVF International Conference on Computer Vision (ICCV) (Seoul: IEEE). 10.1109/ICCV.2019.00700

[B51] Simonyan K. Zisserman A. (2014). Very deep convolutional networks for large-scale image recognition. arXiv [Preprint]. arXiv:1409.1556. 10.48550/arXiv.1409.1556

[B52] Srivastava R. K. Greff K. Schmidhuber J. (2015). “Training Very Deep Networks,” in Advances in Neural Information Processing Systems, Vol. 28.

[B53] Wang X. Li J. Kuang X. Tan Y.-A. Li J. (2019). The security of machine learning in an adversarial setting: a survey. J. Parallel Distributed Comput. 130, 12–23. 10.1016/j.jpdc.2019.03.003

[B54] Wang Y. Huang G. Song S. Pan X. Xia Y. Wu C.. (2021). Regularizing deep networks with semantic data augmentation. IEEE Trans. Pattern Anal. Mach. Intell. 44, 3733–3748. 10.1109/TPAMI.2021.3052951 33476265

[B55] Wei J. Zou K. (2019). “EDA: easy data augmentation techniques for boosting performance on text classification tasks,” in Proceedings of the 2019 Conference on Empirical Methods in Natural Language Processing and the 9th International Joint Conference on Natural Language Processing (EMNLP-IJCNLP) (Hong Kong). 10.18653/v1/D19-1670

[B56] Wu X. Gao C. Lin M. Zang L. Wang Z. Hu S.. (2022). Text smoothing: enhance various data augmentation methods on text classification tasks. arXiv [Preprint]. arXiv:2202.13840. 10.48550/arXiv.2202.13840

[B57] Yao X. Bai Y. Zhang X. Zhang Y. Sun Q. Chen R.. (2022). “PCL: proxy-based contrastive learning for domain generalization,” in Proceedings of the IEEE/CVF Conference on Computer Vision and Pattern Recognition (New Orleans, LA: IEEE), 7097–7107. 10.1109/CVPR52688.2022.00696

[B58] Yu T. Li D. Yang Y. Hospedales T. Xiang T. (2020). “Robust person re-identification by modelling feature uncertainty,” in 2019 IEEE/CVF International Conference on Computer Vision (ICCV) (Seoul: IEEE). 10.1109/ICCV.2019.00064

[B59] Zendrikov D. Solinas S. Indiveri G. (2023). Brain-inspired methods for achieving robust computation in heterogeneous mixed-signal neuromorphic processing systems. Neuromorphic Comput. Eng. 3:034002.

[B60] Zhong Z. Zheng L. Kang G. Li S. Yang Y. (2020). Random erasing data augmentation. Proc. AAAI Conf. Artif. Intell. 34, 13001–13008. 10.1609/aaai.v34i07.7000

[B61] Zhou K. Yang Y. Hospedales T. Xiang T. (2020). “Learning to generate novel domains for domain generalization,” in Computer Vision ECCV 2020: 16th European Conference, Glasgow, UK, August 23–28, 2020, Proceedings, Part XVI (Berlin: Springer), 561–578. 10.1007/978-3-030-58517-4_33

[B62] Zhou K. Yang Y. Qiao Y. Xiang T. (2021). Domain generalization with mixstyle. arXiv [Preprint] arXiv:2104.02008. 10.48550/arXiv.2104.02008

